# Differential co-expression and regulation analyses reveal different mechanisms underlying major depressive disorder and subsyndromal symptomatic depression

**DOI:** 10.1186/s12859-015-0543-y

**Published:** 2015-04-03

**Authors:** Fan Xu, Jing Yang, Jin Chen, Qingyuan Wu, Wei Gong, Jianguo Zhang, Weihua Shao, Jun Mu, Deyu Yang, Yongtao Yang, Zhiwei Li, Peng Xie

**Affiliations:** 1 Department of Neurology, Yongchuan Hospital, Chongqing Medical University, Chongqing, China; 2 Department of Neurology, the First Affiliated Hospital, Chongqing Medical University, No. 1 Youyi Road, Chongqing, 400016 China; 3 Institute of Neuroscience, Chongqing Medical University, and Chongqing Key Laboratory of Neurobiology, Chongqing, China; 4 School of Biotechnology, East China University of Science and Technology, Shanghai, China

**Keywords:** Major depressive disorder, MDD, Subsyndromal symptomatic depression, SSD, Differential co-expression analysis, DCEA, Differential regulation analysis, DRA, Antidepressant, Venlafaxine

## Abstract

**Background:**

Recent depression research has revealed a growing awareness of how to best classify depression into depressive subtypes. Appropriately subtyping depression can lead to identification of subtypes that are more responsive to current pharmacological treatment and aid in separating out depressed patients in which current antidepressants are not particularly effective.

Differential co-expression analysis (DCEA) and differential regulation analysis (DRA) were applied to compare the transcriptomic profiles of peripheral blood lymphocytes from patients with two depressive subtypes: major depressive disorder (MDD) and subsyndromal symptomatic depression (SSD).

**Results:**

Six differentially regulated genes (DRGs) (FOSL1, SRF, JUN, TFAP4, SOX9, and HLF) and 16 transcription factor-to-target differentially co-expressed gene links or pairs (TF2target DCLs) appear to be the key differential factors in MDD; in contrast, one DRG (PATZ1) and eight TF2target DCLs appear to be the key differential factors in SSD. There was no overlap between the MDD target genes and SSD target genes. Venlafaxine (Efexor™, Effexor™) appears to have a significant effect on the gene expression profile of MDD patients but no significant effect on the gene expression profile of SSD patients.

**Conclusion:**

DCEA and DRA revealed no apparent similarities between the differential regulatory processes underlying MDD and SSD. This bioinformatic analysis may provide novel insights that can support future antidepressant R&D efforts.

**Electronic supplementary material:**

The online version of this article (doi:10.1186/s12859-015-0543-y) contains supplementary material, which is available to authorized users.

## Background

Clinicians currently face a large number of patients who suffer from major depressive disorder (MDD, major depression), many of whom live under difficult circumstances and some who finally succumb to suicide. According to 2010 estimates, there were over 298 million cases of MDD globally, with the highest proportion of cases occurring between patients aged 25 and 34 years [1]. Global point prevalence has been relatively constant across time (4.4% across 1990, 2005, and 2010), ranging from 3.7% in North America to 8.6% in South Asia with conflict regions displaying higher prevalences than those with no conflict [1]. Moreover, in 2010, prevalence was higher in females (5.5%) as compared to males (3.2%) [1]. The annual incidence of an episode of MDD followed a similar age and regional pattern to prevalence but was about one and a half times higher, consistent with an average duration of 37.7 weeks [2].

Unfortunately, MDD is often resistant to standard antidepressant medication, and a large percentage of patients respond just as well to placebo [3,4]. As it is unlikely that a polymorphic syndrome like depression reflects a single disease process, recent depression research has revealed a growing awareness of how to best classify depression into depressive subtypes [5]. However, the broad DSM-based diagnosis of MDD does not encourage a search for depressive subtypes that may require their own specific treatments, and most antidepressant trials are commercially-sponsored multicenter studies that aggregate many possible subtypes under an overarching ‘depression’ umbrella for the sake of powering without regard to depressive subtypes [5]. In contrast, the clinical reality is that physicians regularly subtype depression when describing patients to colleagues [5]. Appropriately subtyping depression should lead to identification of subtypes that are more responsive to current pharmacological treatment and aid in separating out depressed patients in which current antidepressants are not highly effective.

One reason for this failure at depression subtyping is our insufficient understanding of the various neurobiological processes underlying depression [6]. An improved understanding of the pathophysiological mechanisms underlying different depressive subtypes should aid antidepressant pharmacological development. For instance, previous studies by our group have applied proteomic, transcriptomic, and metabololomic approaches to better characterize MDD and rodent models of depression [7-11]. Other advanced technologies such as DNA microarrays and next-generation sequencing can allow for rapidly acquiring detailed biochemical information about DNA polymorphisms and transcriptome profiles in different depressive subtypes [12]. These studies collectively show profound biochemical changes in depressed patients, and animal depression models partly corroborate or complement the alterations identified in depressed patients. Transcriptional changes in the course of MDD and the effects of antidepressant drugs on multiple disease-related transcriptional pathways have also been surveyed. The combination of both can unravel additional mechanisms of disease etiology and can provide a ‘bottom-up’ approach for the discovery of novel antidepressant drugs.

One depressive subtype – subsyndromal symptomatic depression (SSD) – has been identified as a transitory depressive state in the depression spectrum. In this study, we hypothesized that the transcriptomic profiles of leukocytes derived from drug-free first-episode SSD patients and MDD patients were significantly different in these two depressive subtypes. Additionally, we hypothesized that these transcriptomic profiles were significantly different before and after treatment with the popular antidepressant venlafaxine (Efexor™, Effexor™). Applying differential co-expression analysis (DCEA) and differential regulation analysis (DRA), the transcriptomic profiles of leukocytes derived from drug-free first-episode SSD patients, MDD patients, and healthy controls were compared using whole-genome cDNA microarrays in order to discover venlafaxine’s mechanism (s) of action in MDD and SSD patients.

## Methods

### Expression dataset

We reutilized the GSE32280 dataset [13], which was published on GEO (http://www.ncbi.nlm.nih.gov/geo/query/acc.cgi?acc=gse32280), to unveil the dysregulated mechanisms in MDD and SSD and explore the differential processes of venlafaxine action on MDD and SSD. The GSE32280 data consists of whole blood gene expression profiles of five SSD patients and eight MDD patients before venlafaxine treatment (hereinafter ‘pre-treatment’) and after venlafaxine treatment (hereinafter ‘post-treatment’) as well as eight healthy controls (Table 1). Raw data were normalized by the RMA method and log2 transformed. After filtering out ambiguous genes, 20283 gene expression values were obtained for all samples.

**Table 1:** **Sample characteristics**

**Group**	**N**	**Biological samples**	**Venlafaxine treatment**	
			**Pre-treatment**	**Post-treatment**
Control	8	Peripheral blood lymphocytes	√	-
MDD	8	Peripheral blood lymphocytes	√	√
SSD	5	Peripheral blood lymphocytes	√	√

### Differential Co-Expression Analysis (DCEA)

In transcriptomics, differential co-expression analysis (DCEA) has emerged as a unique complement to traditional differential expression analysis (DEA) [14]. Rather than merely calculating expression level changes in individual genes as DEA does, DCEA analyzes differences in gene interconnection by calculating the expression correlation changes of gene pairs between two conditions [14-16]. The rationale underlying DCEA is that changes in gene co-expression patterns between contrasting phenotypes (in this case, SSD versus MDD versus healthy control) provide insight into the disrupted regulatory relationships or affected regulatory subnetworks specific to the phenotype (s) of interest.

We utilized the DCGL software package to conduct DCEA, which is a software program released as an R package including two gene filtering functions, three link filtering functions, and five DCEA functions [17,18] In the DCGL software package, differential co-expression profile (DCp) and differential co-expression enrichment (DCe) are designed based on the exact co-expression changes of gene pairs, and thus can differentiate significant co-expression changes from relatively trivial ones and identify co-expression reversal between positive and negative.

For example, for the MDD vs. healthy control comparison, the DCGL package constructed two co-expression networks by calculating gene pair-wise correlation coefficients based on MDD samples and healthy samples. Then, a cutoff for correlation coefficients was applied to filter out some gene pairs and retain the most relevant gene pairs to form two co-expression networks. These two co-expression networks possessed the same topological structure with different correlation coefficients for each gene pair. In these co-expression networks, genes that connected with other genes were deemed to be neighboring genes. Then, DCp was employed to calculate the change between the two co-expression networks that was defined by different co-expression (dC) (see Eq. 1 below). In order to evaluate the statistical significance of dC, DCp implemented a permutation test, in which we shuffled 20283 genes and samples randomly 500 times. Then, we estimated 500 pseudo dCs for every gene using the permuted data. Finally, the *p*-value for each gene could be estimated, and the FDR value could be obtained accordingly. Differentially co-expressed genes (DCGs) were identified by p-value and/or FDR threshold. DCe used the limit fold change (LFC) model [19] to sort out different co-expression gene pairs or differentially co-expressed links (DCLs) and employed a binomial probability model to identify DCGs based on enrichment of DCLs (see Eq. 2 below).

1 $$ d{C}_i(DCp)=\sqrt{\frac{{\left({x}_{i1}-{y}_{i1}\right)}^2+{\left({x}_{i2}-{y}_{i2}\right)}^2+\dots +{\left({x}_{in}-{y}_{in}\right)}^2}{n}} $$

2 $$ d{C}_i(DCe)=P\left({g}_i\right)={\displaystyle \sum_{x={k}_i}^{n_i}{C}_{n_i}^x}{\left(\frac{K}{N}\right)}^x{\left(1-\frac{K}{N}\right)}^{n_i-x} $$

where x_i1_, x_i2_ … x_in_ (y_i1_, y_i2_ … y_in_) denote correlation coefficients between gene i with its n neighbors in condition x (y) and n denoting the number of neighbors. In DCe, N represents the number of links in each co-expression network, K represents the number of DCLs determined by the LFC model. Eq. 2 calculates the enrichment of DCL for gene i with n_i_ links of which k_i_ are DCLs.

### Differential regulation analysis (DRA)

Among the many growing directions of DCEA, there is differential regulation analysis (DRA), which integrates the transcription factor-to-target (TF2target) information to probe upstream regulatory events that account for the observed co-expression changes [20,21]. DRA can unveil the central regulatory network which straightforwardly reflected the differential regulation mechanisms of two contrastive conditions via differential co-expression analysis results. DCGL package was also used in the differential regulation analysis step. The DCGL package contains a library of transcription factors known to regulate the relationships between human target genes termed the transcription factor-to-target library (TF2targetlibrary)*,* which includes 214607 binary tuples involving 215 human transcription factors and 16863 targets [18].

Using this TF2target library, the *DRsort* function of DCGL was used to scrutinize the DCGs and DCLs against the transcription factor-to-target information to highlight a subset of the genes and links that are potentially highly related to the putative differential regulation mechanisms. If a DCG coincides with a transcription factor (TF), it is regarded as a differentially regulated gene (DRG) based on the likelihood that a differential co-expression of this type of DCG could be attributed to disrupted regulatory relationships between the transcription factor and its targets. Hereinafter, ‘TF2target DCLs’ refers to differentially co-expressed gene links or pairs (DCLs) that coincide with transcription factor-to-target relations. Our rationale here is that the disruption of regulatory relations can affect the co-expression links between a regulator and its targets [18].

### Application of DCEA and DRA

To explore the underlying regulatory mechanisms in MDD, we conducted DCEA and DRA to compare the two different MDD states (pre-treatment and post-treatment), pre-treatment MDD versus healthy control, and post-treatment MDD versus healthy control. To explore the effects of venlafaxine therapy on MDD patients, we conducted DCEA and DRA to compare post-treatment MDD versus healthy controls and pre-treatment MDD versus post-treatment MDD. An identical analysis was performed on SSD samples as well. First, we identified DCGs thorough the DCp function, and DCLs and another list of DCGs through the DCe function. Then, in order to avoid lack of individual method, we sorted out true DCGs via overlapping DCp’s DCGs and DCe’s DCGs. DCLs were defined as true DCLs if one of the genes among the DCLs was identified in overlapping DCGs. Finally, the TF2target library was employed to highlight DCGs and DCLs. If a DCG coincided with a TF, it was listed as a DRG. If a DCL coincided with a TF-regulated target gene in the TF2target library, it was listed as a TF2target DCL or DRL.

## Results

### Differential regulatory mechanisms in MDD

**Figure 1:**
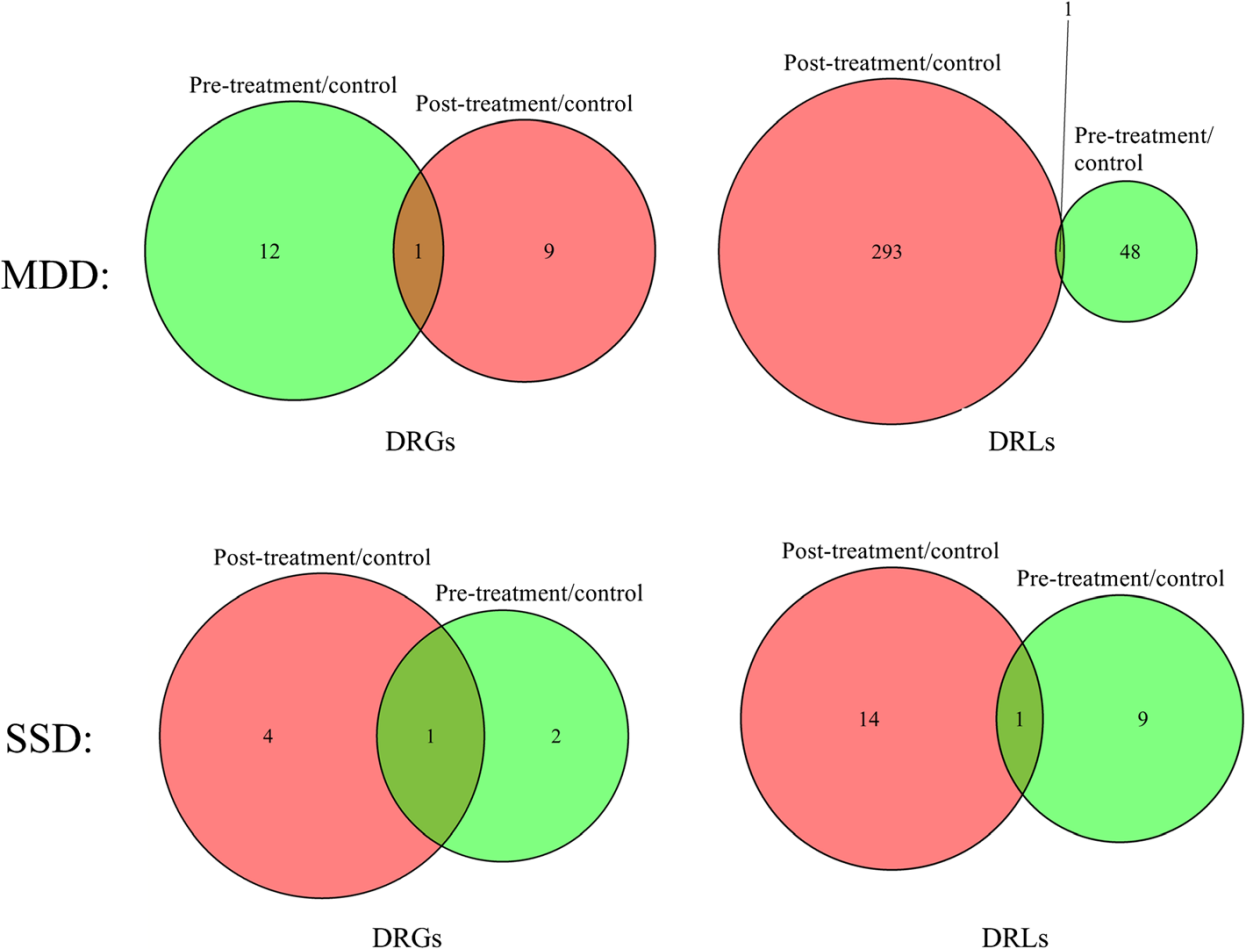
**Venn Diagrams of DRGs and TF2target DCLs in MDD and SSD.** In the MDD panel, DRGs and TF2target DCLs (i.e. DRLs) among pre-treatment MDD versus healthy control (pre-treatment/control), post-treatment MDD versus healthy control (post-treatment/control), and pre-treatment MDD versus post-treatment MDD (pre-treatment/post-treatment). The lime green area of pre-treatment/control, excluding the overlapping brown area of pre-treatment/control with post-treatment/control, indicates the 12 DRGs and 48 DRLs in MDD. In the SSD panel, DRGs and TF2target DCLs (i.e. DRLs) among pre-treatment/control, post-treatment/control, and pre-treatment/post-treatment. The lime green area of pre-treatment/control, excluding the brown overlapping area of pre-treatment/control area with post-treatment/control, indicates the 2 DRGs and 9 DRLs in SSD.

In order to reveal the underlying regulatory mechanisms of non-medicated MDD, samples of pre-treatment MDD versus healthy controls were compared. First, we identified 13 putative DRGs (termed DRG_MDD-pre_ that included FOSL1, FOXL1, MEF2A, HNF1A, IRF1, JUN, SOX9, SRF, TFAP4, TFCP2, TLX2, HLF, and ZNF423) and 49 putative TF2target DCLs (termed DRL_MDD-pre_ that are displayed in Table 2). Furthermore, by comparing post-treatment MDD and healthy control samples, 10 putative DRGs (termed DRG_MDD-pos_ that included BPTF, EP300, FOXI1, IL10, NFKB1, NFYC, NR3C1, TP53, USF2, and FOXL1) and 294 putative TF2target DCLs (termed DRL_MDD-post_ that are shown in Additional file 1: Table S1) were identified. In order to exclude differences within individuals from different samples, the overlapping elements between pre-treatment MDD versus healthy controls and post-treatment MDD versus healthy controls were deemed to indicate differential regulatory information among individuals. Therefore, the expression results that excluded the overlapping differences in co-expression of pre-treatment MDD versus healthy controls were deemed to be the DRGs and TF2target DCLs responsible for differentiating MDD and healthy controls (MDD panel, Figure 1). Therefore, DRG_MDD_ = DRG_MDD − pre_ − (DRG_MDD − pre_ ∩ DRG_MDD − *post*_) were deemed to be the true MDD-related DRGs (termed DRG_MDD_ that included FOSL1, MEF2A, HNF1A, IRF1, JUN, SOX9, SRF, TFAP4, TFCP2, TLX2, HLF, and ZNF423). These 12 true DRG_MDD_ are depicted in Figure 1 by the lime green area of pre-treatment/control that excludes the overlapping brown area of pre-treatment/control with post-treatment/control. Based on the foregoing analysis, these 12 DRG_MDD_ may be involved in the pathological processes underlying MDD.

**Table 2:** **Identification of 49 TF2target DCLs through comparing MDD pre-treatment and healthy control samples**

**NO.**	**TF**	**Target**	**p. DCG**	**Cor.1**	**Cor.2**	**Type**	**NO.**	**TF**	**Target**	**p. DCG**	**Cor.1**	**Cor.2**	**Type**
1	AHR	TLE4^*^	6.77E–23	0.94	0.14	0	26	NFYA	PANK2^*^	5.37E–05	–0.13	0.97	1
2	ZEB1	ATP12A^*^	3.56E-73	-0.54	0.95	1	27	MRPL36	NR4A1^*^	1.34E–08	0.88	–0.94	1
3	FOXL1^*^	CBX6	0.014521	0.95	0.13	0	28	NF1	NUPL2^*^	1.86E–35	0.91	0.10	0
4	CEBPB	PLEKHM1^*^	0.000574	0.18	0.94	0	29	ZEB1	OTOF^*^	2.62E–07	0.06	-0.96	1
5	CREB1	TGM1^*^	0.001644	0.87	0.05	0	30	HAND1	PITX2^*^	5.83E–06	-0.90	-0.01	0
6	CREB1	CROCCP2^*^	5.02E–09	0.92	0.07	0	31	PLAU	LNP1^*^	2.00E–19	-0.01	-0.92	0
7	PATZ1	DYRK2^*^	2.79E–16	-0.88	-0.02	0	32	PSG1	YRDC^*^	6.27E–11	0.11	0.93	0
8	FOS	FEZ2^*^	0.000107	0.03	0.96	0	33	RFX1	SNORA21^*^	3.87E–38	0.98	-0.05	1
9	MYCN	FLRT2^*^	9.32E–05	0.29	-0.98	1	34	RFX1	LOC283481^*^	3.34E–55	0.96	-0.33	1
**10**	**FOSL1** ^*^	**HIVEP3**	**6.37E–06**	**0.94**	**0.02**	**0**	**35**	**SOX9** ^*^	**FLAD1**	**8.34E–09**	**-0.92**	**-0.10**	**0**
**11**	**FOSL1** ^*^	**DSCAML1**	**6.37E–06**	**0.91**	**0.16**	**0**	36	SREBF1	PHOX2B^*^	0.002661	0.89	0.06	0
12	GATA1	COL19A1^*^	9.63E–10	0.12	0.93	0	**37**	**SRF** ^*^	**C4orf7**	**1.23E–18**	**-0.96**	**0.74**	**1**
**13**	**JUN** ^*^	**GRIA4**	**4.30E–10**	**-0.60**	**0.97**	**1**	**38**	**SRF** ^*^	**SHMT1**	**1.23E─18**	**-0.92**	**-0.12**	**0**
**14**	**SRF** ^*^	**HCG18**	**1.23E─18**	**-0.91**	**-0.14**	**0**	39	STAT5B	TRAK2^*^	1.42E─06	0.45	-0.95	1
**15**	**HLF** ^*^	**LRRTM4**	**0.022779**	**0.92**	**0.18**	**0**	40	TCF3	C11orf61^*^	0.003455	-0.87	-0.10	0
16	E2F5	ID1^*^	0.008474	-0.88	-0.11	0	**41**	**TFAP4** ^*^	**BSCL2**	**3.03E–16**	**-0.06**	**-0.94**	**0**
**17**	**JUN** ^*^	**ZNF830**	**4.30E–10**	**-0.09**	**-0.93**	**0**	**42**	**TLX2** ^*^	**C3orf70** ^*^		**0.03**	**-0.97**	**1**
**18**	**JUN** ^*^	**SPOP**	**4.30E–10**	**-0.11**	**-0.95**	**0**	43	FOXL1^*^	TOB1	0.014521	-0.88	-0.11	0
**19**	**JUN** ^*^	**GAK**	**4.30E–10**	**-0.43**	**0.96**	**1**	44	TLX2^*^	TSPAN8	6.53E–06	-0.94	-0.11	0
**20**	**JUN** ^*^	**CHURC1**	**4.30E–10**	**0.51**	**-0.95**	**1**	45	CREB1	VPS26A^*^	6.76E–05	0.92	0.05	0
21	PAX5	KCTD7^*^	1.14E–05	0.97	-0.54	1	**46**	**SRF** ^*^	**VWA3A**	**1.23E–18**	**-0.89**	**-0.08**	**0**
22	JUN^*^	KLF6	4.30E–10	0.89	0.12	0	47	YY1	ZNF518A^*^	1.93E–119	0.90	0.04	0
23	MZF1	MID1^*^	0.006234	0.96	-0.28	1	48	POU2F1	ZNF518A^*^	1.93E–119	0.98	-0.15	1
24	MZF1	EPHB2^*^	0.012953	-0.93	-0.13	0	49	ZSCAN1	KRT13^*^	4.80E–10	0.89	0.15	0
**25**	**SRF** ^*^	**NDUFA1**	**1.23E–18**	**0.88**	**0.12**	**0**							

^*^Denotes DCG in each TF2target DCL. Some rows were discarded (strikethrough) since they are individual basis. Rows in bold represent the 16 key-DRL_MDD_. The p. DCG column denotes the *p*-value of DCG for each TF2target DCL; the “Cor.1” and “Cor.2” columns represent the correlation coefficients of the two respective conditions. The “Type” column reflects the mode of regulation with “1” denoting a positive/negative regulation in disease samples switched to a negative/positive regulation in control samples and “0” denoting no change in regulation.

**Figure 2:**
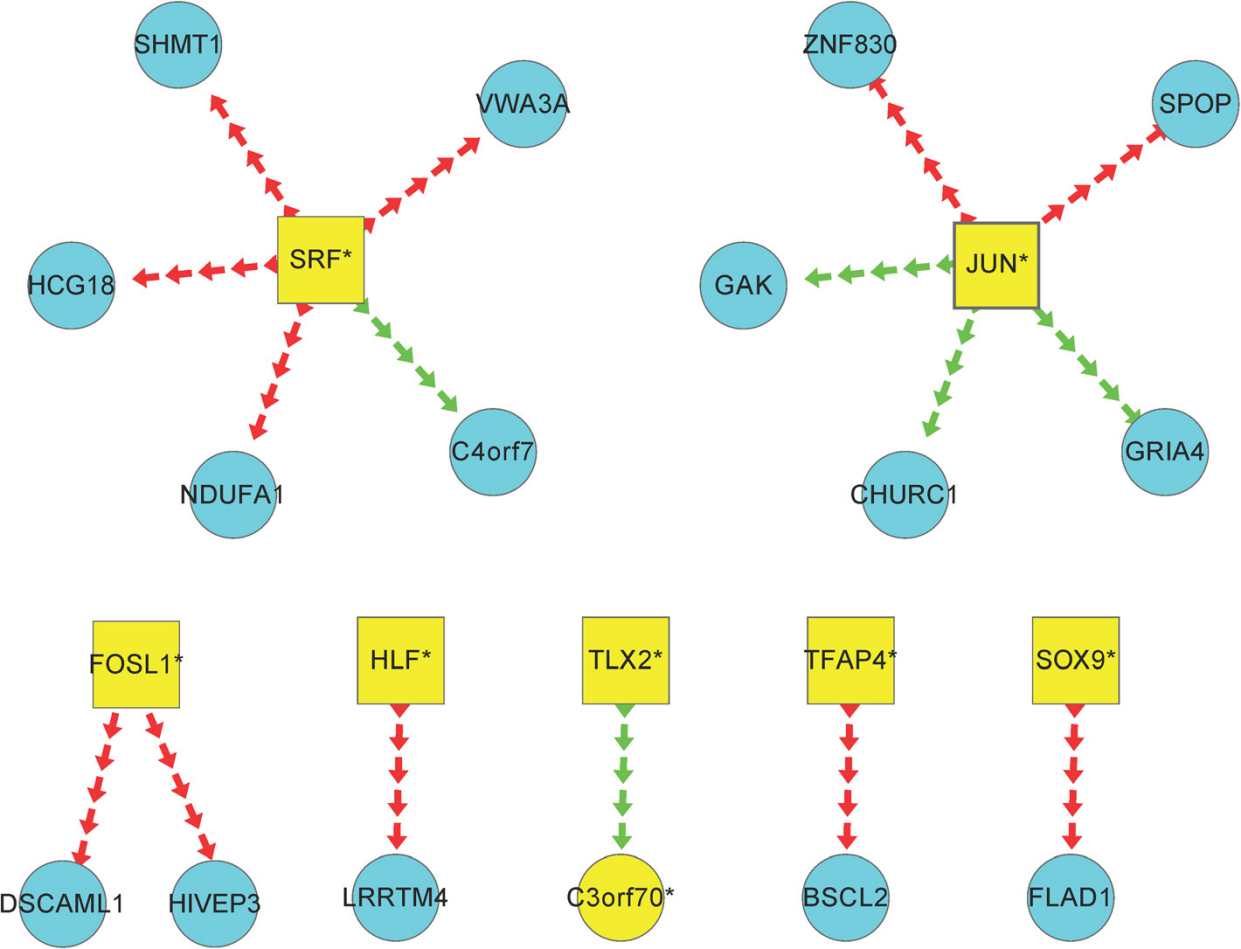
**Key TF2target DCLs in MDD.** Every line with an arrow represents a differential regulatory relationship in MDD. Squares represent transcription factors (yellow = differential, blue = non-differential), circles represent target genes (yellow = differential, blue = non-differential), and lines represent the action of transcription factors on target genes (green = positive regulation in disease samples transformed to negative regulation in control samples or vice-versa, red = unchanged regulation).

According to the same logical analysis applied to DRG, we identified 48 true MDD-related TF2target DCLs (i.e., DRLs) according to the following formula: DRL_MDD_ = DRL_MDD − pre_ − (DRL_MDD − pre_ ∩ DRL_MDD − *post*_). The 48 TF2target DCLs contained 33 transcription factors and 47 target genes (Table 2 except striked-through rows). By overlapping the 12 DRG_MDD_ with the 48 DRL_MDD_, six key DRGs (termed key-DRG_MDD_ that included FOSL1, SRF, JUN, TFAP4, SOX9, and HLF) and 16 TF2target DCLs (termed key-DRL_MDD_) appear to be the key differential factors in MDD (Figure 2). GO analysis revealed that the six key-DRG_MDD_ are involved in RNA metabolism (SRF, JUN, HLF, and FOSL1) and developmental processes (SRF and JUN) (Additional file 1: Table S3, Figure S1). Moreover, four key-DRG_MDD_ (SRF, JUN, HLF, and FOSL1) localize to the nucleus and are enriched for transcription regulatory activity (Additional file 1: Table S3, Figure S1).

### Potential mechanism of venlafaxine in MDD

**Figure 3:**
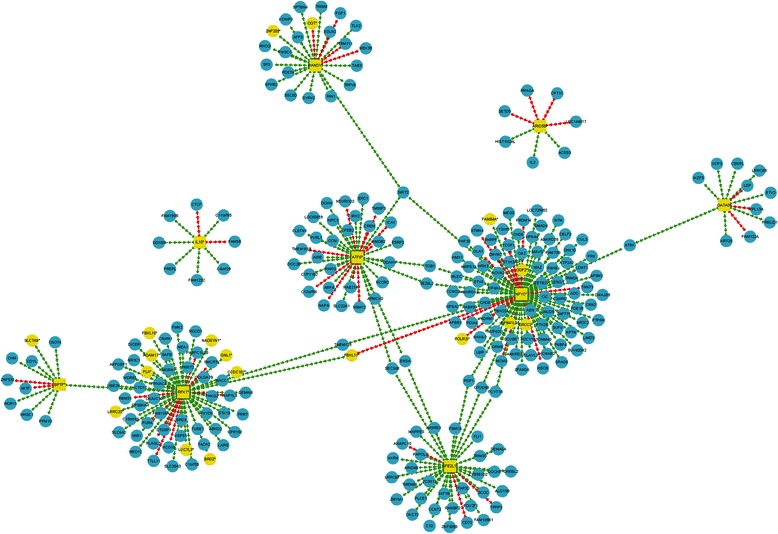
**Key TF2target DCLs of Venlafaxine in MDD.** Nine differentially regulated genes (yellow squares) display the action of venlafaxine in MDD. Squares represent transcription factors (yellow = differential, blue = non-differential), circles represent target genes (yellow = differential, blue = non-differential), and lines represent the action of transcription factors on target genes (green = positive regulation in disease samples transformed to negative regulation in control samples or vice-versa, red = unchanged regulation).

In order to discover the pharmacological mechanism (s) of venlafaxine in MDD, samples from the same MDD patients pre-treatment and post-treatment were compared. Consequently, a total of nine DRGs and 532 TF2target DCLs were discovered. The 532 TF2target DCLs contained 65 transcription factors and 466 target genes (Additional file 1: Table S2). Nine differential regulatory transcription factors, which participated in TF2target DCLs, appear to be the key differential regulatory relation pairs reflecting the pharmacological action of venlafaxine in MDD (Figure 3).

### Differential regulatory mechanism in SSD

**Figure 4:**
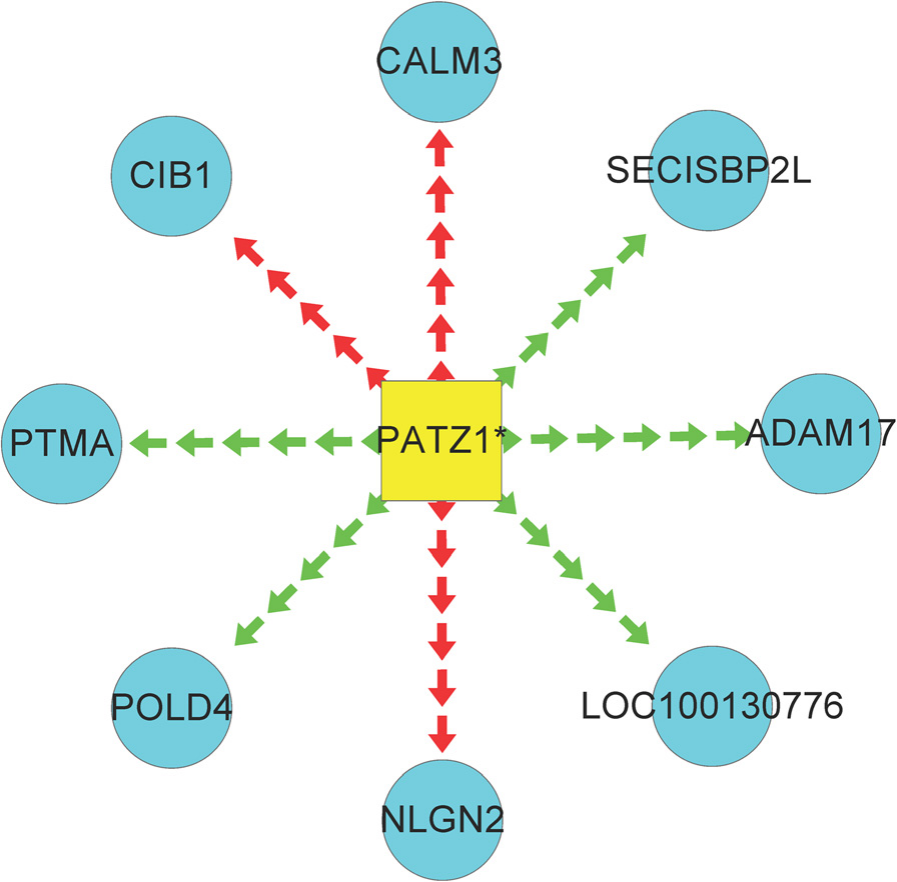
**Key TF2target DCLs in SSD.** Eight TF2target DCLs showing the differential regulatory mechanisms in SSD. Squares represent transcription factors (yellow = differential, blue = non-differential), circles represent target genes (yellow = differential, blue = non-differential), and lines represent the action of transcription factors on target genes (green = positive regulation in disease samples transformed to negative regulation in control samples or vice-versa, red = unchanged regulation).

Similar to the analysis conducted in MDD, the DCGL software package identified three putative DRGs (termed DRG_SSD-pre_ that included FOSB, PATZ1, and TFAP4) and 10 putative TF2target DCLs (termed DRL_SSD-pre_ that are displayed in Table 3) between pre-treatment SSD and healthy control samples. A comparison of post-treatment SSD with healthy control samples yielded five DRGs (termed DRG_SSD-post_ that included EGR1, IRF1, MYOD1, SOX5, and TFAP4) and 15 TF2target DCLs (termed DRL_SSD-post_). After exclusion on an individual basis, i.e., and DRL_SSD_ = DRL_SSD − pre_ − (DRL_SSD − pre_ ∩ DRL_SSD − *post*_), two DRGs (DRG_SSD_ = PATZ1 and FOSB) and nine DRL_SSD_ appear to be associated with SSD. The nine DRL_SSD_ are indicated by the lime green area that excludes the overlapping brown area (SSD panel, Figure 1) and listed in Table 3 (except striked-through rows). Applying a similar analysis to that used in MDD by overlapping the 2 DRG_SSD_ with the 9 DRL_SSD_, we determined one key-DRG_SSD_ (PATZ1) and 8 key-DRL_SSD_ in SSD (Figure 4).

**Table 3:** **Identification of 10 TF2taraget DCLs through comparing SSD pre-treatment and healthy control samples**

**No.**	**TF**	**Target**	**p. DCG**	**cor.1**	**cor.2**	**Type**
**1**	**PATZ1** ^*^	**ADAM17**	**0.018596**	**-0.71**	**0.97**	**1**
**2**	**PATZ1** ^*^	**CALM3**	**0.018596**	**0.03**	**0.94**	**0**
**3**	**PATZ1** ^*^	**CIB1**	**0.018596**	**0.03**	**0.93**	**0**
4	TP53	FUS^*^	0.001496	-0.47	0.97	*1*
**5**	**PATZ1** ^*^	**LOC100130776**	**0.018596**	**0.54**	**-0.97**	**1**
6	CBFB	MACROD2^*^	0.029467	-0.53	0.97	1
**7**	**PATZ1** ^*^	**NLGN2**	**0.018596**	**-0.07**	**-0.94**	**0**
**8**	**PATZ1** ^*^	**SECISBP2L**	**0.018596**	**-0.68**	**0.97**	**1**
**9**	**PATZ1** ^*^	**POLD4**	**0.018596**	**-0.18**	**0.97**	**1**
**10**	**PATZ1** ^*^	**PTMA**	**0.018596**	**-0.37**	**0.97**	**1**

^*^Denotes DCG in each TF2target DCL. Some rows were discarded (strikethrough) since they are individual basis. Rows in bold represent the 8 key-DRL_SSD_. The p. DCG column denotes the *p*-value of DCG for each TF2target DCL; the “Cor.1” and “Cor.2” columns represent the correlation coefficients of the two respective conditions. The “Type” column reflects the mode of regulation with “1” denoting a positive/negative regulation in disease samples switched to a negative/positive regulation in control samples and “0” denoting no change in regulation.

### Potential mechanism of venlafaxine in SSD

No DRG or TF2target DCLs were identified between SSD pre- and post-treatment. Therefore, the expression profiles between pre- and post-treatment SSD samples were not significantly different; thus, venlaxafine appears to have no significant effect on the gene expression profile of SSD patients.

## Discussion

With regard to the differential regulatory mechanism (s) underlying MDD, six key-DRG_MDD_ (FOSL1, SRF, JUN, TFAP4, SOX9, and HLF) and 16 key-DRL_MDD_ (TF2target DCLs) appear to be the key differential factors in MDD (Table 2). FOSL1 has been previously associated with addiction, depression, and anxiety [22]. SRF and JUN (c-JUN) are functionally involved in the MAPK signaling pathway (Additional file 1: Figure S2) [13]. GO analysis revealed that the six key-DRG_SSD_ are involved in RNA metabolism (SRF, JUN, HLF, and FOSL1) and developmental processes (SRF and JUN) (Additional file 1: Table S3, Figure S1). Moreover, four key-DRG_MDD_ (SRF, JUN, HLF, and FOSL1) localize to the nucleus and are enriched for transcription regulatory activity (Additional file 1: Table S3, Figure S1).

With regard to the differential regulatory mechanism (s) underlying SSD, one key-DRG_SSD_ (PATZ1) and eight key-DRL_SSD_ (TF2target DCLs) appear to be the key differential factors in SSD (Table 3). Unfortunately, after an extensive literature search, we found no previous studies showing any association between these genes and SSD, which may be attributed to the paucity of research on SSD. These initial findings can open an entirely new field of investigation on the underlying regulatory mechanism (s) of SSD.

As for a potential mechanism (s) underlying venlafaxine in MDD and SSD, nine DRGs and 532 TF2target DCLs were responsible for distinguishing pre-treatment and post-treatment MDD samples (Additional file 1: Table S2). However, no DRGs or TF2target DCLs were found between pre-treatment and post-treatment SSD samples, suggesting that venlaxafine appears to have a significant effect on the gene expression profile of MDD patients but no significant effect on the gene expression profile of SSD patients.

### Different mechanisms underlying MDD and SSD

DCEA and DRA revealed no similarities between the differential regulatory processes underlying MDD and SSD. Specifically, six DRGs (FOSL1, SRF, JUN, TFAP4, SOX9, and HLF) and 16 TF2target DCLs appear to be the key differential factors in MDD; in contrast, one DRG (PATZ1) and eight TF2target DCLs appear to be the key differential factors in SSD (Tables 2, 3). Moreover, there was no overlap in MDD target genes and SSD target genes. While there were 62 downstream target genes for MDD or SSD, there was no overlap in MDD target genes and SSD target genes. In addition, via the Wilcoxon test, we found that the distance between TFs and target genes in SSD were significantly larger than those in MDD (*p* = 0.02). Therefore, although MDD appears to include more key-DRG_MDD_ and key-DRL_MDD_ as compared to the number of key-DRG_SSD_ and key-DRL_SSD_ in SSD, the extent of differential gene regulation in MDD was lower than that in SSD. Based on this evidence, we hypothesize that MDD involves preferential influence to a wider set of genes, while SSD involves preferential influence of gene regulatory elements.

With regard to venlafaxine’s mode of action in MDD, nine transcription factors (ARID5B, ATF6, BPTF, GATA3, HAND1, IL10, NFE2L1, NFYC, and RFX1) were found to act on 256 target genes. In contrast, no differences between pre-treatment and post-treatment SSD were identified, suggesting that venlafaxine has no significant effect on the gene expression profile of SSD patients. Therefore, as the aforementioned evidence reveals that MDD and SSD display completely different underlying mechanisms, we presume that venlaxafine has no significant effect on SSD patients due to the different underlying mechanisms of the two depressive subtypes. In future studies, conducting a similar analysis involving additional antidepressants would provide additional evidence to support this view and may provide insight into other antidepressant therapies or potential drug targets that may be more efficacious for SSD patients.

### Numerous hypotheses for MDD

The pathoetiology underlying MDD remains largely unknown [23]. MDD can spontaneously develop but often follows a traumatic emotional experience or can be a symptom of other diseases, most often neurological (e.g., stroke, multiple sclerosis, and Parkinson’s disease) or endocrine (e.g., Cushing’s disease and hypothyroidism). MDD can also be triggered by pharmacological agents or drug abuse [24]. Different mechanisms have been proposed to explain the pathophysiological basis of MDD from a neurobiological point of view [25,26] These hypotheses include monoaminergic deficiency, hypothalamic-pituitary-adrenal (HPA) axis dysregulation [27-29], neurogenetic and neurotrophic-growth factor impairment, metabolic disturbances, circadian rhythm desynchronization, and inappropriate stimulation of the immune system [25,26,30]. This multiplicity of hypotheses can be explained by the fact that several of them are intertwined rather than being mutually exclusive [27-30]. Moreover, it is probable that different endophenotypes correlate with different neurobiological adaptations. From this vantage point, a lack of criteria in selecting appropriate patients may contribute to the difficulties experienced in the quest for new therapies that rely on non-monoamine mechanisms of action.

### Lighting the future for antidepressant R&D

The efficacy of available antidepressant therapies has been demonstrated with respect to placebo, especially in cases of severe depression [31]. Nevertheless, a relatively large number of patients still fail to respond to conventional treatment [32]. Moreover, a number of symptoms may not be adequately resolved in patients that experience an overall positive therapeutic response. In addition, although the safety profile of newer antidepressant agents is quite superior to older drugs, the incidence of side effects, such as sexual dysfunction, may cause therapy discontinuation, especially in younger patients. Therefore, considerable efforts have been dedicated to developing therapeutic agents that address novel neurobiological targets with the hope of overcoming the aforementioned issues [33,34].

Unfortunately, approaches aimed at identifying therapies based on different mechanisms have not been successful [35], and pharmaceutical companies are disengaging from this disease area, which has been perceived as highly risky. One reason is our insufficient understanding of the neurobiological basis of MDD [26,30]. As discussed earlier, MDD is likely a constellation of merging disease states that can be split into endophenotypes [27]. Several lines of evidence support the notion that the occurrence of environmental challenges, often in the form of stressful experiences, needs to be associated with a pre-existing genetic predisposition to bring about the disease [36]. The bioinformatic analysis in this report provides reliable evidence that can support future antidepressant R&D efforts.

### Study limitations

First, the sample size of the current study was limited; thus, the low power of this study limits the applicability of our findings. Second, this study only employed two methods – DCEA and DRA -- to analyze the expression profiles of the samples. Although this did provide novel evidence that can aid in pharmacological target development, no biological experiments were performed to validate the findings. Therefore, further biological experiments with larger sample sizes are required to validate the current findings. Third, it would have been more insightful to measure clinical response to venlaxafine in terms of improvements in depression rating scales in lieu of assessing differential expression pre- and post-treatment without regard to clinical response. Unfortunately, there were no clinical response data reported in the previously published studies. Therefore, for our future research on this topic, we will collect patient samples from our hospital and record the clinical responses in terms of improvements in depression rating scales.

## Conclusions

Here, we applied DCEA and DRA in comparing the transcriptomic profiles of peripheral blood lymphocytes from MDD and SSD patients, which revealed no apparent similarities between the differential regulatory processes underlying MDD and SSD. Six DRGs (FOSL1, SRF, JUN, TFAP4, SOX9, and HLF) and 16 TF2target DCLs appear to be the key differential factors in MDD; in contrast, one DRG (PATZ1) and eight TF2target DCLs appear to be the key differential factors in SSD. Moreover, there appears to be no overlap between MDD target genes and SSD target genes. In addition, venlaxafine appears to have a significant effect on the gene expression profile of MDD patients but appears to have no significant effect on the gene expression profile of SSD patients. This bioinformatic analysis may provide novel insights that can support future antidepressant R&D efforts.

## Additional files

Additional file 1: Table S1. The 294 TF2target DCLs in post-treatment MDD versus healthy controls. **Table S2.** The 532 TF2target DCLs of venlafaxine action in MDD. **Table S3.** GO analysis of the twelve DRGs in MDD. **Figure S1.** GO analysis of the twelve DRGs in MDD. **Figure S2.** Participation of the two key DRGs for MDD (SRK and JUN) in the MAPK signaling pathway. Full schematic of the MAPK signaling pathway showing SRF and JUN (c-JUN) (highlighted in red).
