# Supplementary material for: Differential co-expression and regulation analyses reveal different mechanisms underlying major depressive disorder and subsyndromal symptomatic depression
Source: BMC Bioinformatics. 2015 Apr 3;16:112. doi: 10.1186/s12859-015-0543-y (PMC4434877; doi:10.1186/s12859-015-0543-y)
Supplement: Additional file 1: Table S1. — The 294 TF2target DCLs in post-treatment MDD versus healthy controls. Table S2. The 532 TF2target DCLs of venlafaxine action in MDD. Table S3. GO analysis of the twelve DRGs in MDD. Figure S1. GO analysis of the twelve DRGs in MDD. Figure S2. Participation of the two key DRGs for MDD (SRK and JUN) in the MAPK signaling pathway. Full schematic of the MAPK signaling pathway showing SRF and JUN (c-JUN) (highlighted in red). [file 12859_2015_543_MOESM1_ESM.docx]

# Additional file

**Table S1. The 294 TF2target DCLs in Post-Treatment MDD Versus Healthy Controls**

| **Transcription factor** | **Target gene** | **DCG** |
| --- | --- | --- |
| AHR | KDM5A | KDM5A |
| AHR | KIAA2018 | KIAA2018 |
| AHR | MKLN1 | MKLN1 |
| AHR | NFYC | NFYC |
| AHR | CHIC2 | CHIC2 |
| AHR | KCNAB3 | KCNAB3 |
| AHR | RAB2A | RAB2A |
| ARID5B | ABI1 | ABI1 |
| ARNT | CCNO | CCNO |
| ATF6 | ENSA | ENSA |
| ATF6 | ZBTB20 | ZBTB20 |
| ATF6 | CYP11B2 | CYP11B2 |
| BACH2 | SH3BP1 | SH3BP1 |
| BPTF | PPM1D | BPTF |
| BPTF | PHEX | PHEX; BPTF |
| CBFB | KDM4A | KDM4A |
| CEBPA | EXT1 | EXT1 |
| CEBPA | LOC643923 | LOC643923 |
| CEBPA | IFI16 | IFI16 |
| CEBPA | SH3PXD2A | SH3PXD2A |
| CREB1 | BAIAP2L2 | BAIAP2L2 |
| CREB1 | COPS7A | COPS7A |
| CREB1 | ATAD2B | ATAD2B |
| CREB1 | CDH2 | CDH2 |
| CREB1 | DLEU2 | DLEU2 |
| CREB1 | DMRTA2 | DMRTA2 |
| CREB1 | TP53 | TP53 |
| CREB1 | WAPAL | WAPAL |
| CREB1 | CRTC1 | CRTC1 |
| CREB1 | DLG4 | DLG4 |
| CREB1 | GDAP1L1 | GDAP1L1 |
| CREB1 | MMP28 | MMP28 |
| CREB1 | SCYL2 | SCYL2 |
| CREB1 | SLC25A11 | SLC25A11 |
| CREB1 | TCF25 | TCF25 |
| CREB1 | YIPF6 | YIPF6 |
| E2F1 | DYRK1A | DYRK1A |
| E2F1 | EP300 | EP300 |
| E2F1 | KDM5A | KDM5A |
| E2F1 | SPOCK3 | SPOCK3 |
| E2F1 | ERBB2IP | ERBB2IP |
| E2F1 | GOLGA2 | GOLGA2 |
| E2F1 | ZNF771 | ZNF771 |
| E2F4 | DYRK1A | DYRK1A |
| EP300 | ADM2 | EP300 |
| EP300 | B3GNT2 | EP300 |
| EP300 | CAMKV | EP300 |
| EP300 | CDC73 | EP300 |
| EP300 | CDK12 | EP300 |
| EP300 | DOC2B | EP300 |
| EP300 | EDA | EP300 |
| EP300 | ELF2 | EP300 |
| EP300 | ARIH1 | EP300 |
| EP300 | PHF20 | EP300 |
| EP300 | WDR62 | EP300; WDR62 |
| EP300 | FAM188B | EP300 |
| EP300 | FOXF2 | EP300 |
| EP300 | GATAD2A | EP300 |
| EP300 | HMGXB3 | HMGXB3; EP300 |
| EP300 | HOXB8 | EP300 |
| EP300 | INO80E | INO80E; EP300 |
| EP300 | IRX5 | EP300 |
| EP300 | ITPR1 | EP300 |
| EP300 | KCNC2 | EP300 |
| EP300 | KRT17 | EP300 |
| EP300 | LRRC57 | EP300 |
| EP300 | LY6G6D | EP300 |
| EP300 | MAPK8IP2 | EP300 |
| EP300 | PCNX | EP300 |
| EP300 | PITX3 | EP300 |
| EP300 | RASSF2 | EP300 |
| EP300 | RBM27 | EP300 |
| EP300 | SEMA3A | EP300 |
| EP300 | SNCAIP | EP300 |
| EP300 | TBCC | EP300 |
| EP300 | TRIM63 | EP300 |
| EP300 | USP34 | EP300 |
| EP300 | ZNF646 | EP300 |
| ESR1 | GFRA2 | GFRA2 |
| FOXD3 | PDZD7 | PDZD7 |
| FOXD3 | HMP19 | HMP19 |
| FOXF2 | FOXF2 | CELF2 |
| FOXI1 | NFIA | FOXI1 |
| FOXJ2 | EFNA1 | EFNA1 |
| FOXL1 | ADAMTS8 | FOXL1 |
| FOXL1 | C5orf43 | FOXL1 |
| FOXL1 | COL4A3BP | FOXL1 |
| FOXL1 | DAK | FOXL1 |
| FOXL1 | EPS15 | FOXL1 |
| FOXL1 | CRIM1 | FOXL1 |
| FOXL1 | ELAVL4 | FOXL1 |
| FOXL1 | FGF1 | FOXL1 |
| FOXL1 | ING3 | FOXL1 |
| FOXL1 | LOC253044 | FOXL1 |
| FOXL1 | MAPK13 | FOXL1 |
| FOXL1 | NR0B1 | FOXL1 |
| FOXL1 | PAPOLG | FOXL1 |
| FOXL1 | SCRN3 | FOXL1 |
| FOXL1 | VAPB | FOXL1 |
| FOXL1 | SP8 | FOXL1 |
| FOXL1 | TAOK3 | FOXL1 |
| FOXO1 | CELF2 | CELF2 |
| FOXO1 | SH3D19 | SH3D19 |
| FOXO1 | ICA1 | ICA1 |
| FOXO4 | FHOD3 | FHOD3 |
| FOXO4 | PDZD7 | PDZD7 |
| FOXO4 | RMND5A | RMND5A |
| GATA1 | LGI1 | LGI1 |
| HOXA3 | CEP164 | CEP164 |
| HOXA3 | KIAA2018 | KIAA2018 |
| HOXA3 | NFYC | NFYC |
| HSF2 | SMURF2 | SMURF2 |
| IL10 | C11orf95 | IL10 |
| IL10 | C4orf29 | IL10 |
| IL10 | CRAMP1L | IL10 |
| IL10 | FAM133A | IL10 |
| IL10 | DDX50 | IL10 |
| IL10 | FAM122C | IL10 |
| IL10 | FAM190B | IL10 |
| IL10 | PREPL | IL10 |
| IRF2 | ANKRD17 | ANKRD17 |
| MAX | MTA2 | MTA2 |
| MIA3 | ANK1 | ANK1 |
| MIA3 | FBXO7 | FBXO7 |
| MYOD1 | ZNHIT2 | ZNHIT2 |
| NF1 | CNOT3 | CNOT3 |
| NF1 | EP300 | EP300 |
| NF1 | SH3D19 | SH3D19 |
| NF1 | GAB1 | GAB1 |
| NF1 | HSPG2 | HSPG2 |
| NFKB1 | GATA4 | NFKB1 |
| NFYB | SLC25A11 | SLC25A11 |
| NFYC | ANKRD28 | NFYC |
| NFYC | ATP2A2 | NFYC |
| NFYC | CUL3 | NFYC |
| NFYC | DHX15 | NFYC |
| NFYC | FAM73B | NFYC |
| NFYC | FAM84A | NFYC |
| NFYC | GRM8 | NFYC |
| NFYC | HMGB2 | NFYC |
| NFYC | IGSF9B | NFYC |
| NFYC | IL6ST | NFYC |
| NFYC | ING2 | NFYC |
| NFYC | KPNA4 | NFYC |
| NFYC | LENG1 | NFYC |
| NFYC | LIMD2 | NFYC |
| NFYC | LOC100499177 | NFYC |
| NFYC | MAVS | NFYC |
| NFYC | MLF1 | NFYC |
| NFYC | DNAJB9 | NFYC |
| NFYC | ELAVL3 | NFYC |
| NFYC | ETV4 | NFYC |
| NFYC | HRH3 | NFYC |
| NFYC | MEG3 | NFYC |
| NFYC | SIRT2 | NFYC |
| NFYC | NKX6-2 | NFYC |
| NFYC | NOVA2 | NFYC |
| NFYC | NTM | NTM; NFYC |
| NFYC | NUMBL | NFYC |
| NFYC | OTUD6B | NFYC |
| NFYC | PDE1B | NFYC |
| NFYC | PPP1R15B | NFYC |
| NFYC | SEZ6L2 | NFYC |
| NFYC | SMAD6 | NFYC |
| NFYC | SUV420H2 | NFYC |
| NFYC | TOB1 | NFYC |
| NFYC | ZFAND6 | NFYC |
| NFYC | JMJD8 | NFYC |
| NFYC | LBR | NFYC |
| NFYC | CCNG2 | NFYC |
| NFYC | MACROD2 | NFYC |
| NFYC | OAT | NFYC |
| NFYC | PRX | NFYC |
| NFYC | PTPRH | NFYC |
| NFYC | RPS6KL1 | NFYC |
| NFYC | SCUBE1 | NFYC |
| NFYC | SEC23B | NFYC |
| NFYC | TMEM129 | TMEM129; NFYC |
| NR3C1 | ADAM11 | NR3C1 |
| NR3C1 | ANXA9 | NR3C1 |
| NR3C1 | CAMK2A | NR3C1 |
| NR3C1 | CBLB | NR3C1 |
| NR3C1 | HNRNPR | NR3C1 |
| NR3C1 | IL17RC | NR3C1 |
| NR3C1 | BTBD16 | NR3C1 |
| NR3C1 | C11orf36 | NR3C1 |
| NR3C1 | COPE | NR3C1 |
| NR3C1 | GPR113 | NR3C1 |
| NR3C1 | HIF3A | NR3C1 |
| NR3C1 | MARCKSL1 | NR3C1 |
| NR3C1 | NR5A2 | NR3C1 |
| NR3C1 | SH3D19 | NR3C1; SH3D19 |
| NR3C1 | ZNF644 | NR3C1 |
| NR3C1 | NUS1 | NR3C1 |
| NR3C1 | RBM7 | NR3C1 |
| NR3C1 | RPS6KA3 | NR3C1 |
| NR3C1 | USP2 | NR3C1 |
| NR3C1 | YARS2 | NR3C1 |
| NR3C1 | ZNF771 | ZNF771; NR3C1 |
| NR3C1 | HSPB6 | NR3C1 |
| NR3C1 | CLP1 | NR3C1 |
| NR3C1 | SLC4A7 | NR3C1 |
| PATZ1 | KIAA1033 | KIAA1033 |
| PATZ1 | CASKIN1 | CASKIN1 |
| PATZ1 | UBE4B | UBE4B |
| PATZ1 | PLSCR3 | PLSCR3 |
| PAX3 | BAIAP2L2 | BAIAP2L2 |
| PAX3 | SHISA4 | SHISA4 |
| POU3F2 | SLC25A23 | SLC25A23 |
| PSG1 | ARPC4 | ARPC4 |
| PSG1 | EEFSEC | EEFSEC |
| PSG1 | ATAD2B | ATAD2B |
| PSG1 | ATP6V0E1 | ATP6V0E1 |
| PSG1 | MBD3 | MBD3 |
| PSG1 | NR3C1 | NR3C1 |
| PSG1 | NUTF2 | NUTF2 |
| PSG1 | RAD23A | RAD23A |
| PSG1 | SLC16A8 | SLC16A8 |
| REL | KRT83 | KRT83 |
| REL | REL | WDR62 |
| RFX1 | DOK1 | DOK1 |
| RFX1 | FAM160A2 | FAM160A2 |
| RFX1 | PRKCG | PRKCG |
| RFX1 | RQCD1 | RQCD1 |
| RFX1 | RNF25 | RNF25 |
| SP1 | PPP1R12C | PPP1R12C |
| STAT5A | HGF | HGF |
| STAT6 | HGF | HGF |
| TCF3 | FAM86A | FAM86A |
| TLX2 | DMRTA2 | DMRTA2 |
| TLX2 | FOXN3 | FOXN3 |
| TLX2 | MKLN1 | MKLN1 |
| TLX2 | LTB4R2 | LTB4R2 |
| TP53 | ASF1A | TP53 |
| TP53 | C12orf26 | TP53 |
| TP53 | C9orf16 | TP53 |
| TP53 | CCNG1 | TP53 |
| TP53 | CREBL2 | TP53 |
| TP53 | CTCF | TP53 |
| TP53 | DCAF17 | TP53 |
| TP53 | DLG2 | TP53 |
| TP53 | DLG4 | DLG4; TP53 |
| TP53 | DUOXA2 | TP53 |
| TP53 | EED | TP53 |
| TP53 | EID2 | TP53 |
| TP53 | FAM3A | FAM3A; TP53 |
| TP53 | MAPK8IP3 | TP53 |
| TP53 | MDFI | TP53 |
| TP53 | NDUFA13 | NDUFA13; TP53 |
| TP53 | PIK3CG | TP53 |
| TP53 | PM20D2 | TP53 |
| TP53 | POC1B | TP53 |
| TP53 | SHC3 | TP53 |
| TP53 | SLC7A10 | SLC7A10; TP53 |
| TP53 | TCF4 | TP53 |
| TP53 | TMPO | TP53 |
| TP53 | COX8A | TP53; COX8A |
| TP53 | HIPK1 | TP53 |
| TP53 | KANK2 | TP53 |
| TP53 | KLC3 | TP53 |
| TP53 | NEK4 | TP53 |
| TP53 | PFN1 | TP53; PFN1 |
| TP53 | ST8SIA4 | TP53 |
| TP53 | ZNF277 | TP53 |
| TP53 | UTP18 | TP53 |
| TP53 | ZNF580 | ZNF580; TP53 |
| TP53 | ZNF654 | TP53 |
| USF1 | KIAA1033 | KIAA1033 |
| USF1 | PSMF1 | PSMF1 |
| USF2 | GNPTG | USF2 |
| USF2 | RBM15B | USF2 |
| USF2 | RGS3 | USF2 |
| USF2 | S100A6 | USF2 |
| USF2 | SASH3 | SASH3; USF2 |
| USF2 | SCAF1 | USF2 |
| USF2 | GIT2 | USF2 |
| USF2 | PDE5A | USF2 |
| USF2 | ZNF740 | USF2 |
| USF2 | ASGR1 | USF2 |
| USF2 | CLN3 | USF2 |
| YY1 | HNRNPU | HNRNPU |
| YY1 | PPM1B | PPM1B |
| YY1 | SAR1A | SAR1A |
| YY1 | SLC25A28 | SLC25A28 |
| YY1 | ATAD2B | ATAD2B |
| YY1 | NFYC | NFYC |
| YY1 | NLGN2 | NLGN2 |
| YY1 | NRP2 | NRP2 |
| ZEB1 | CACNG4 | CACNG4 |
| ZEB1 | ATP12A | ATP12A |
| ZEB1 | EP300 | EP300 |
| ZIC3 | MBD3 | MBD3 |
| ZSCAN1 | PHEX | PHEX |
| ZSCAN1 | DLEU2 | DLEU2 |

**Table S2. The 532 TF2target DCLs of Venlafaxine Action in MDD**

| **Transcription factor** | **Target gene** | **DCG** |
| --- | --- | --- |
| AHR | ARHGDIA | ARHGDIA |
| AHR | ARIH2 | ARIH2 |
| AHR | BAZ2A | BAZ2A |
| AHR | BLOC1S1 | BLOC1S1 |
| AHR | CIB1 | CIB1 |
| AHR | CIRBP | CIRBP |
| AHR | DCAF5 | DCAF5 |
| AHR | HDAC9 | HDAC9 |
| AHR | HNRNPU | HNRNPU |
| AHR | NAA15 | NAA15 |
| AHR | NBL1 | NBL1 |
| AHR | NFYC | NFYC |
| AHR | PPP3CA | PPP3CA |
| AHR | RBM15B | RBM15B |
| AHR | TERF2IP | TERF2IP |
| AHR | WNK1 | WNK1 |
| AHR | ZMYND11 | ZMYND11 |
| ATF6 | DDAH1 | ATF6 |
| ATF6 | ENSA | ATF6 |
| ATF6 | KIFC3 | ATF6 |
| ATF6 | LOC92659 | ATF6 |
| ATF6 | NEUROD2 | ATF6 |
| ATF6 | SIRT2 | ATF6 |
| BPTF | CDYL | BPTF |
| BPTF | CHM | BPTF |
| BPTF | CNOT4 | BPTF |
| BPTF | MITF | BPTF |
| BPTF | PPM1D | BPTF |
| BPTF | RFX1 | BPTF; RFX1 |
| CEBPA | ARRB2 | ARRB2 |
| CREB1 | ARIH1 | ARIH1 |
| E2F5 | CCT3 | CCT3 |
| EN1 | PRPF3 | PRPF3 |
| EP300 | SON | SON |
| FOXF2 | ARID5B | ARID5B |
| FOXO1 | PDCD4 | PDCD4 |
| FOXO1 | PPP3CA | PPP3CA |
| FOXO4 | NAV2 | NAV2 |
| GATA1 | SYNCRIP | SYNCRIP |
| GATA3 | LEP | GATA3 |
| GCGR | FSCN2 | FSCN2 |
| HAND1 | DYRK2 | HAND1 |
| HAND1 | EPHB2 | HAND1 |
| HAND1 | KCNIP3 | HAND1 |
| HAND1 | OGT | HAND1; OGT |
| HAND1 | PDE7A | HAND1 |
| HAND1 | PIK3CG | HAND1 |
| HAND1 | RHOG | HAND1 |
| HAND1 | RIN1 | HAND1 |
| HAND1 | SIRT2 | HAND1 |
| HAND1 | SP3 | HAND1 |
| HAND1 | TLK2 | HAND1 |
| HAND1 | TRPV6 | HAND1 |
| HNF1A | P2RY6 | P2RY6 |
| HNF1A | TLK1 | TLK1 |
| HOXA3 | NFYC | NFYC |
| HOXA5 | TNFSF11 | TNFSF11 |
| IL10 | CTCF | IL10 |
| IL10 | DDX50 | IL10 |
| IL10 | FAM122C | IL10 |
| IL10 | FAM190B | IL10 |
| MAX | PSME3 | PSME3 |
| MYCN | HMG20B | HMG20B |
| MYOD1 | SYNCRIP | SYNCRIP |
| NF1 | CNOT3 | CNOT3 |
| NF1 | DHH | DHH |
| NF1 | NTN5 | NTN5 |
| NF1 | SMARCA4 | SMARCA4 |
| NFE2L1 | MSRB3 | NFE2L1 |
| NFE2L1 | PLCE1 | NFE2L1 |
| NFE2L1 | SEMA6A | NFE2L1 |
| NFE2L1 | ZNF385B | NFE2L1 |
| NFYB | SLC25A11 | SLC25A11 |
| NFYC | ADC | NFYC |
| NFYC | CHD6 | NFYC |
| NFYC | DNAJB9 | NFYC |
| NFYC | ELAVL3 | NFYC |
| NFYC | ETV4 | NFYC |
| NFYC | HRH3 | NFYC |
| NFYC | MEG3 | NFYC |
| NFYC | SIRT2 | NFYC |
| NFYC | SPAG9 | NFYC |
| NR3C1 | TBCB | TBCB |
| PATZ1 | BAZ2A | BAZ2A |
| PATZ1 | DDX5 | DDX5 |
| PATZ1 | HDAC2 | HDAC2 |
| PELP1 | LOC643923 | LOC643923 |
| PLAU | CYTH2 | CYTH2 |
| POU2F1 | BAZ2A | BAZ2A |
| POU2F1 | GALNT1 | GALNT1 |
| POU2F1 | PDE6D | PDE6D |
| POU2F1 | PTPN9 | PTPN9 |
| POU2F1 | R3HDM2 | R3HDM2 |
| POU2F1 | RBM15B | RBM15B |
| POU2F1 | SMS | SMS |
| POU2F1 | ZMYND11 | ZMYND11 |
| POU3F2 | NAV2 | NAV2 |
| REL | NDUFS1 | NDUFS1 |
| RFX1 | ABHD2 | RFX1 |
| RFX1 | ADAM17 | RFX1; ADAM17 |
| RFX1 | ARFGEF1 | RFX1 |
| RFX1 | BRD3 | RFX1 |
| RFX1 | C1orf55 | RFX1 |
| RFX1 | DICER1 | RFX1 |
| RFX1 | FMN2 | RFX1 |
| RFX1 | GNL1 | RFX1; GNL1 |
| RFX1 | HCG18 | RFX1 |
| RFX1 | KCTD17 | RFX1 |
| RFX1 | LRRC23 | RFX1; LRRC23 |
| RFX1 | LUC7L3 | RFX1; LUC7L3 |
| RFX1 | MED12 | RFX1 |
| RFX1 | MIB1 | RFX1 |
| RFX1 | MORN1 | RFX1 |
| RFX1 | NADSYN1 | RFX1; NADSYN1 |
| RFX1 | NAP1L3 | RFX1 |
| RFX1 | NR3C1 | RFX1 |
| RFX1 | PABPC1L2B | RFX1 |
| RFX1 | PHKG2 | RFX1 |
| RFX1 | PLAGL2 | RFX1 |
| RFX1 | PRKACA | RFX1 |
| RFX1 | PURA | RFX1 |
| RFX1 | RBMS1 | RFX1 |
| RFX1 | RPS6KA4 | RFX1 |
| RFX1 | RQCD1 | RFX1 |
| RFX1 | SAFB | RFX1 |
| RFX1 | SMCR7L | RFX1 |
| RFX1 | STK19 | RFX1 |
| RFX1 | TAOK2 | RFX1 |
| RFX1 | UBE2N | RFX1 |
| RFX1 | USP31 | RFX1 |
| TCF3 | HTRA2 | HTRA2 |
| TCF3 | NBL1 | NBL1 |
| TFAP4 | DNAJB1 | DNAJB1 |
| TLX2 | DMRTA2 | DMRTA2 |
| TLX2 | FSCN2 | FSCN2 |
| TLX2 | HTRA2 | HTRA2 |
| TLX2 | RNPS1 | RNPS1 |
| TP53 | FBXO7 | FBXO7 |
| TP53 | PFN1 | PFN1 |
| TP53 | WDR1 | WDR1 |
| USF1 | ASXL2 | ASXL2 |
| USF1 | CDK6 | CDK6 |
| USF1 | ISG20L2 | ISG20L2 |
| USF1 | MSI2 | MSI2 |
| USF1 | PIK3R1 | PIK3R1 |
| USF1 | UBE2H | UBE2H |
| XBP1 | ARSK | ARSK |
| YY1 | NAP1L1 | NAP1L1 |
| YY1 | NFYC | NFYC |
| YY1 | SREK1IP1 | SREK1IP1 |
| YY1 | SYNCRIP | SYNCRIP |
| ATF6 | ARF4 | ATF6 |
| ATF6 | ERC1 | ATF6 |
| ATF6 | SEC24B | ATF6 |
| ATF6 | TOB1 | ATF6 |
| BAX | PDE1A | PDE1A |
| FOXL1 | MAPK13 | MAPK13 |
| FOXL1 | PPP3CA | PPP3CA |
| GATA3 | ATRX | GATA3 |
| GATA3 | CENPL | GATA3 |
| GATA3 | RPL13A | GATA3 |
| GATA3 | UCP3 | GATA3 |
| HAND1 | AFF3 | HAND1 |
| HAND1 | FGF1 | HAND1 |
| HAND1 | TAB3 | HAND1 |
| HAND1 | TRMT11 | HAND1 |
| HAND1 | ZNF205 | HAND1; ZNF205 |
| HSF2 | HTRA2 | HTRA2 |
| MRPL36 | ANAPC2 | ANAPC2 |
| MRPL36 | NIPBL | NIPBL |
| MRPL36 | SCN1B | SCN1B |
| MRPL36 | UPP1 | UPP1 |
| MRPL36 | WDR45 | WDR45 |
| NFE2L1 | C1D | NFE2L1 |
| NFE2L1 | PAPOLG | NFE2L1 |
| NFYC | CCNG2 | NFYC |
| NFYC | CELF2 | NFYC |
| PAX5 | PTPN9 | PTPN9 |
| PLAU | SEC31A | SEC31A |
| PLAU | SLC35B1 | SLC35B1 |
| PLAU | UBXN1 | UBXN1 |
| TLX2 | GTF2E2 | GTF2E2 |
| USF1 | C5orf4 | C5orf4 |
| USF1 | ZMYND8 | ZMYND8 |
| SP1 | ACIN1 | ACIN1 |
| ARID5B | ACSS3 | ARID5B |
| SP1 | ADAM17 | ADAM17 |
| NFYC | ADCY10 | NFYC |
| NFYC | ADRM1 | NFYC |
| AHR | AKIRIN1 | AKIRIN1 |
| NFE2L1 | ALG10B | NFE2L1 |
| NFYC | ANKRD12 | NFYC |
| NFYC | ANKRD28 | NFYC |
| NFYC | ANKRD37 | NFYC |
| SP1 | AP2A1 | AP2A1 |
| POU2F1 | ARHGAP18 | ARHGAP18 |
| SP1 | ARHGEF1 | ARHGEF1 |
| NFE2L1 | ARID4B | NFE2L1 |
| YY1 | ARIH1 | ARIH1 |
| SP1 | ARRB2 | ARRB2 |
| NFYC | ATP2A2 | NFYC |
| POU2F1 | BCAS3 | BCAS3 |
| SP1 | BCL7C | BCL7C |
| RFX1 | BRD2 | BRD2; RFX1 |
| IL10 | C11orf95 | IL10 |
| NFYC | C12orf5 | NFYC |
| RELA | C17orf49 | C17orf49 |
| PLAU | C17orf61 | C17orf61 |
| POU2F1 | C17orf61 | C17orf61 |
| RFX1 | C19orf43 | RFX1 |
| IL10 | C4orf29 | IL10 |
| RFX1 | C9orf41 | RFX1 |
| CREB1 | CACNG4 | CACNG4 |
| TP53 | CARM1 | CARM1 |
| CBFB | CASP2 | CASP2 |
| RFX1 | CCDC107 | CCDC107; RFX1 |
| AHR | CCDC130 | CCDC130 |
| NFE2L1 | CCNT2 | NFE2L1 |
| NFE2L1 | CD72 | NFE2L1 |
| FOXO4 | CDH10 | CDH10 |
| NFYC | CHD8 | NFYC |
| AHR | CHIC2 | CHIC2 |
| CREB1 | CHURC1 | CHURC1 |
| NFYC | CIC | NFYC |
| AHR | CMIP | CMIP |
| FOXO4 | CMIP | CMIP |
| NFYC | CNN2 | NFYC |
| AHR | CNOT3 | CNOT3 |
| YY1 | CNOT3 | CNOT3 |
| E2F1 | CNOT6 | CNOT6 |
| NR3C1 | CPNE1 | CPNE1 |
| NFE2L1 | CREBL2 | NFE2L1 |
| ATF6 | CREM | ATF6 |
| PATZ1 | CSRNP2 | CSRNP2 |
| RFX1 | CTDSP1 | RFX1 |
| NFYC | CUL3 | NFYC |
| NFE2L1 | CYB561D2 | NFE2L1 |
| SP1 | CYTH2 | CYTH2 |
| SP1 | DDX5 | DDX5 |
| NFYC | DHX15 | NFYC |
| RFX1 | DNAJC10 | RFX1 |
| SP1 | DNAJC4 | DNAJC4 |
| NFE2L1 | DOCK9 | NFE2L1 |
| SP1 | DVL3 | DVL3 |
| E2F1 | DYRK1A | DYRK1A |
| NR3C1 | EFHD2 | EFHD2 |
| HAND1 | EGLN2 | HAND1 |
| NFYC | EIF4A1 | NFYC |
| NFE2L1 | ENSA | NFE2L1 |
| CEBPA | EPB41L5 | EPB41L5 |
| ATF6 | ESRP2 | ATF6 |
| TLX2 | ESRRG | ESRRG |
| CREB1 | FAF2 | FAF2 |
| NFE2L1 | FAM108B1 | NFE2L1 |
| GATA3 | FAM122A | GATA3 |
| RFX1 | FAM126A | RFX1 |
| MYOD1 | FAM168A | FAM168A |
| PATZ1 | FAM168A | FAM168A |
| USF2 | FAM168B | FAM168B |
| SP1 | FAM50A | FAM50A |
| NFYC | FAM73B | NFYC |
| NFYC | FAM84A | FAM84A; NFYC |
| AHR | FBXL15 | FBXL15 |
| RFX1 | FBXL15 | FBXL15; RFX1 |
| NFYC | FBXL17 | FBXL17; NFYC |
| RFX1 | FBXL17 | FBXL17; RFX1 |
| NFYC | FBXO24 | NFYC |
| SP1 | FIS1 | FIS1 |
| NFE2L1 | FLI1 | NFE2L1 |
| RFX1 | FLII | FLII; RFX1 |
| CREB1 | FOXN3 | FOXN3 |
| NR3C1 | FSCN2 | FSCN2 |
| RFX1 | FXYD5 | RFX1 |
| SP1 | GBA2 | GBA2 |
| RFX1 | GEMIN8 | RFX1 |
| SP1 | GLTP | GLTP |
| SP1 | GNL1 | GNL1 |
| RFX1 | GOLGA7B | RFX1 |
| RFX1 | GPR153 | RFX1 |
| NFYC | GRM8 | NFYC |
| AHR | HAT1 | HAT1 |
| FOXD3 | HEPACAM2 | HEPACAM2 |
| NFYC | HIST1H2AK | NFYC |
| ATF6 | HMGB2 | ATF6 |
| PTK7 | HNF1B | HNF1B |
| NFYC | HSCB | NFYC |
| GATA3 | IKZF5 | GATA3 |
| ARID5B | IL2 | ARID5B |
| FOXJ2 | ING2 | ING2 |
| YY1 | INO80E | INO80E |
| NFYC | KCNAB2 | NFYC |
| HNF1A | KIAA0182 | KIAA0182 |
| NR3C1 | KIAA0182 | KIAA0182 |
| SP1 | KIAA1967 | KIAA1967 |
| NFE2L1 | KIF1B | NFE2L1 |
| NFYC | KPNA2 | NFYC |
| NFYC | KPNA4 | NFYC |
| RFX1 | LARS | RFX1 |
| NFYC | LCMT1 | NFYC |
| AHR | LDB1 | LDB1 |
| NR3C1 | LDB1 | LDB1 |
| SP1 | LDB1 | LDB1 |
| NFYC | LENG1 | NFYC |
| NFYC | LIMD2 | NFYC |
| MAX | LOC100144603 | LOC100144603 |
| USF1 | LOC100144603 | LOC100144603 |
| NFYC | LOC729852 | NFYC |
| NFE2L1 | LRRC69 | NFE2L1 |
| YY1 | LSM14A | LSM14A |
| PTK7 | LTBP4 | LTBP4 |
| MZF1 | LUC7L2 | LUC7L2 |
| SP1 | MAD2L2 | MAD2L2 |
| TP53 | MAD2L2 | MAD2L2 |
| NFE2L1 | MAPRE3 | NFE2L1 |
| E2F1 | MASP1 | MASP1 |
| NFYC | MAVS | NFYC |
| NFYC | MAZ | NFYC |
| RFX1 | MEA1 | RFX1 |
| SP1 | METRNL | METRNL |
| HAND1 | MEX3B | HAND1 |
| NFYC | MLEC | NFYC |
| SP1 | MLL4 | MLL4 |
| CREB1 | MOAP1 | MOAP1 |
| YY1 | MRO | MRO |
| POU2F1 | MRPL54 | MRPL54 |
| NFYC | MRPS12 | NFYC |
| AHR | MTF2 | MTF2 |
| RFX1 | NACC1 | RFX1 |
| SP1 | NDRG1 | NDRG1 |
| TP53 | NDUFA13 | NDUFA13 |
| YY1 | NDUFS1 | NDUFS1 |
| CREB1 | NECAP2 | NECAP2 |
| NFYC | NKX6-2 | NFYC |
| TP53 | NOP16 | NOP16 |
| NFYC | NOVA2 | NFYC |
| NFYC | NPTN | NFYC |
| NFYC | NR2C2 | NFYC |
| NF1 | NR2F2 | NR2F2 |
| NFYC | NTM | NFYC |
| NFYC | NUP62CL | NFYC |
| SP1 | NUTF2 | NUTF2 |
| NFYC | ODF2 | ODF2; NFYC |
| NFYC | OLA1 | NFYC |
| TP53 | OTUD3 | OTUD3 |
| NFE2L1 | OTUD6B | NFE2L1 |
| NFYC | OTUD6B | NFYC |
| NFYC | P4HB | NFYC |
| STAT5B | PAF1 | PAF1 |
| NFE2L1 | PCYT1B | NFE2L1 |
| NFYC | PCYT1B | NFYC |
| NFYC | PDE1B | NFYC |
| AHR | PDIA3 | PDIA3 |
| NFYC | PDIA4 | NFYC |
| NFE2L1 | PDP1 | NFE2L1 |
| NFYC | PDP1 | NFYC |
| SP1 | PFN1 | PFN1 |
| NFYC | PHF20 | NFYC |
| E2F1 | PHOX2A | PHOX2A |
| PAX3 | PHOX2A | PHOX2A |
| SRF | PLEKHJ1 | PLEKHJ1 |
| PLAU | PLSCR3 | PLSCR3 |
| EP300 | PNKP | PNKP |
| NFYC | POLR2I | POLR2I; NFYC |
| NFE2L1 | POU2F1 | NFE2L1 |
| PLAU | PPIG | PPIG |
| AHR | PPP2CA | PPP2CA |
| POU2F1 | PPPDE2 | PPPDE2 |
| AHR | PQBP1 | PQBP1 |
| IL10 | PREPL | IL10 |
| RFX1 | PRR7 | RFX1 |
| RFX1 | PRRT2 | RFX1 |
| NFE2L1 | PSMC6 | NFE2L1 |
| NFYC | PTK2B | NFYC |
| STAT1 | PTPN6 | PTPN6 |
| ATF6 | PVRL1 | ATF6 |
| AHR | RAB2A | RAB2A |
| PLAU | RAB2A | RAB2A |
| EN1 | RAB2B | RAB2B |
| NFYC | RAB40C | NFYC |
| SP1 | RAB5B | RAB5B |
| NFE2L1 | RANBP2 | NFE2L1 |
| ATF6 | RBM12 | ATF6 |
| USF2 | RBM15B | RBM15B |
| EP300 | RBM27 | RBM27 |
| SP1 | RBM38 | RBM38 |
| ATF6 | RCOR2 | ATF6 |
| CEBPA | REV1 | REV1 |
| CREB1 | RMND5A | RMND5A |
| FOXO4 | RMND5A | RMND5A |
| TLX2 | RNF115 | RNF115 |
| ARID5B | RRAGA | ARID5B |
| NR3C1 | RWDD1 | RWDD1 |
| E2F1 | SCARF2 | SCARF2 |
| NFE2L1 | SCOC | NFE2L1 |
| NFE2L1 | SEC24B | NFE2L1 |
| YY1 | SEC31A | SEC31A |
| PATZ1 | SETD1B | SETD1B |
| ARID5B | SETD5 | ARID5B |
| ATF6 | SEZ6L2 | ATF6 |
| NFYC | SEZ6L2 | NFYC |
| AHR | SLC25A28 | SLC25A28 |
| SP1 | SLC25A28 | SLC25A28 |
| RFX1 | SLC35A3 | RFX1 |
| RFX1 | SLC6A2 | RFX1 |
| NFYC | SMAD6 | NFYC |
| AHR | SPHK2 | SPHK2 |
| HAND1 | SPTBN4 | HAND1 |
| YY1 | SRSF3 | SRSF3 |
| HAND1 | SSC5D | HAND1 |
| CREB1 | STIM2 | STIM2 |
| NFYC | STMN4 | NFYC |
| SP1 | STX4 | STX4 |
| NFYC | SUFU | NFYC |
| NFYC | SUV420H2 | NFYC |
| AHR | TAF6 | TAF6 |
| CREB1 | TCF25 | TCF25 |
| CUX1 | TES | TES |
| NFE2L1 | TFAP2D | NFE2L1 |
| CBFB | TFDP2 | TFDP2 |
| CBFB | TIAL1 | TIAL1 |
| AHR | TIMM50 | TIMM50 |
| SP1 | TMBIM6 | TMBIM6 |
| RFX1 | TMEM129 | RFX1 |
| SP1 | TMEM219 | TMEM219 |
| NFE2L1 | TMEM66 | NFE2L1 |
| NFYC | TOB1 | NFYC |
| NFYC | TOMM40 | NFYC |
| NFE2L1 | TPPP3 | NFE2L1 |
| NFYC | TRIM24 | NFYC |
| NFE2L1 | TRIM33 | NFE2L1 |
| HAND1 | TRIM8 | HAND1 |
| EN1 | TSHZ1 | TSHZ1 |
| RFX1 | TTLL11 | RFX1 |
| PLAU | TTYH3 | TTYH3 |
| SP1 | TTYH3 | TTYH3 |
| RFX1 | TUBE1 | RFX1 |
| POU2F1 | UBE2H | UBE2H |
| SP1 | UBE2M | UBE2M |
| SP1 | UBXN4 | UBXN4 |
| CEBPA | UFD1L | UFD1L |
| PAX3 | VPS37B | VPS37B |
| RFX1 | WDR83 | RFX1 |
| PATZ1 | WWP1 | WWP1 |
| NFE2L1 | XKR4 | NFE2L1 |
| NFYC | ZBTB22 | NFYC |
| AHR | ZDHHC12 | ZDHHC12 |
| NFYC | ZFAND6 | NFYC |
| NFE2L1 | ZMYM1 | NFE2L1 |
| NFYC | ZMYM2 | NFYC |
| BPTF | ZNF532 | BPTF |
| POU2F1 | ZNF609 | ZNF609 |
| NFYC | ZNF775 | NFYC |
| PTK7 | ZSCAN29 | ZSCAN29 |
| NFYC | AEN | NFYC |
| ATF6 | AIRE | ATF6 |
| NFE2L1 | ANAPC10 | NFE2L1 |
| MAFK | ANAPC2 | ANAPC2 |
| NFYC | AP3M2 | NFYC |
| NFYC | APBB2 | NFYC |
| RELA | ARID5A | ARID5A |
| ATF6 | ARMCX3 | ATF6 |
| NF1 | ARRDC1 | ARRDC1 |
| NFYC | ATRX | NFYC |
| PLAU | AUP1 | AUP1 |
| JUNB | BRD4 | BRD4 |
| ATF6 | C12orf56 | ATF6 |
| NFYC | C2orf42 | NFYC |
| PLAU | C6orf72 | C6orf72 |
| HSF2 | CACNG4 | CACNG4 |
| ATF6 | CCNI | ATF6 |
| ATF6 | CLSTN3 | ATF6 |
| ATF6 | CPEB2 | ATF6 |
| ARID5B | CPT1C | ARID5B |
| PLAU | CSDC2 | CSDC2 |
| ATF6 | CYP11B2 | ATF6 |
| PLAU | DNTTIP1 | DNTTIP1 |
| ATF6 | DOC2B | ATF6 |
| ATF6 | DOHH | ATF6 |
| NFYC | EPB41L5 | EPB41L5; NFYC |
| NFYC | ERCC2 | ERCC2; NFYC |
| GATA3 | ETV2 | GATA3 |
| IL10 | FAM5B | IL10 |
| HSF2 | FLT4 | FLT4 |
| NFYC | FNBP4 | NFYC |
| PLAU | GNL3 | GNL3 |
| ARID5B | HIST1H2AL | ARID5B |
| NFYC | HMX1 | NFYC |
| ATF6 | ICA1 | ATF6 |
| PLAU | INO80E | INO80E |
| ATF6 | IRX2 | ATF6 |
| NFYC | JMJD8 | NFYC |
| GATA3 | KRT25 | GATA3 |
| NFYC | LBR | NFYC |
| ARID5B | LOC144817 | ARID5B |
| GATA3 | LRRC8B | GATA3 |
| RELA | MAP3K8 | MAP3K8 |
| NFYC | MCM3 | NFYC |
| PLAU | MRPL54 | MRPL54 |
| GCGR | MRPS9 | MRPS9 |
| NR3C1 | MRPS9 | MRPS9 |
| ATF6 | NAPA | ATF6 |
| NFYC | OAT | NFYC |
| NFE2L1 | OXCT2 | NFE2L1 |
| E2F4 | PHOX2A | PHOX2A |
| NFYC | PRDM14 | NFYC |
| GATA3 | PRELID1 | GATA3 |
| NFYC | PRX | NFYC |
| NFYC | PTPRH | NFYC |
| PLAU | PYY | PYY |
| ATF6 | RAB27A | ATF6 |
| ATF6 | RARG | ATF6 |
| XBP1 | RFX1 | RFX1 |
| NFYC | SCUBE1 | NFYC |
| PLAU | SECISBP2 | SECISBP2 |
| ATF6 | SLC22A1 | ATF6 |
| PAX5 | SLC35F1 | SLC35F1 |
| BPTF | SLC7A9 | SLC7A9; BPTF |
| NFYC | SNRPF | NFYC |
| PLAU | TCF25 | TCF25 |
| NFYC | TCOF1 | NFYC |
| TBP | TFDP2 | TFDP2 |
| ATF6 | TM9SF3 | ATF6 |
| NFYC | TMEM129 | NFYC |
| ATF6 | TMEM161B | ATF6 |
| PLAU | TMEM179B | TMEM179B |
| NFYC | TNNT3 | NFYC |
| MRPL36 | TRAPPC1 | TRAPPC1 |
| PTK7 | USH1C | USH1C |
| BPTF | WDR11 | BPTF |
| BPTF | WHSC1 | BPTF |
| HSF2 | YY1AP1 | YY1AP1 |
| NFE2L1 | ZC3H13 | NFE2L1 |

**Table S3. GO Analysis of the Twelve DRGs in MDD**

**Biological processes**

| **Gene** | **Process** | **Count** | ***P*-value** |
| --- | --- | --- | --- |
| JUN | Cell cycle and proliferation | 1 | 0.488157041 |
| HNF1A | Stress response | 1 | 0.456630106 |
| HNF1A | Transport | 1 | 0.738936398 |
| MEF2A, IRF1, HNF1A, SRF, JUN, TLX2 | Developmental processes | 6 | 0.000470537 |
| JUN | Protein metabolism | 1 | 0.787153161 |
| MEF2A, IRF1, HNF1A, SRF, JUN, HLF, TLX2, FOSL1 | RNA metabolism | 8 | 8.23E-07 |
| IRF1, HNF1A | Other metabolic processes | 2 | 0.440934935 |
| HNF1A, TLX2 | Cell organization and biogenesis | 2 | 0.257494314 |
| HNF1A | Cell-cell signaling | 1 | 0.160145211 |
| HNF1A | Signal transduction | 1 | 0.887777923 |
| MEF2A | Death | 1 | 0.355351915 |
| MEF2A, HNF1A, SRF, JUN, HLF, FOSL1 | Other biological processes | 6 | 0.074062423 |

**Cell components**

| **Gene** | **Component** | **Count** | ***P*-value** |
| --- | --- | --- | --- |
| MEF2A, IRF1, HNF1A, SRF, JUN, HLF, TLX2, FOSL1 | Nucleus | 8 | 2.05E-05 |
| SOX9, TFAP4, TFCP2, ZNF423 | Other cellular component | 4 | 0.727513 |

**Molecular functions**

| **Gene** | **Function** | **Count** | ***P*-value** |
| --- | --- | --- | --- |
| MEF2A, IRF1, HNF1A, SRF, JUN, HLF, TLX2, FOSL1 | Transcription regulatory activity | 8 | 3.53E-10 |
| SOX9, TFAP4, TFCP2, ZNF423 | Other molecular function | 4 | 0.436 |

**Figure S1. GO Analysis of the Twelve DRGs in MDD**

**
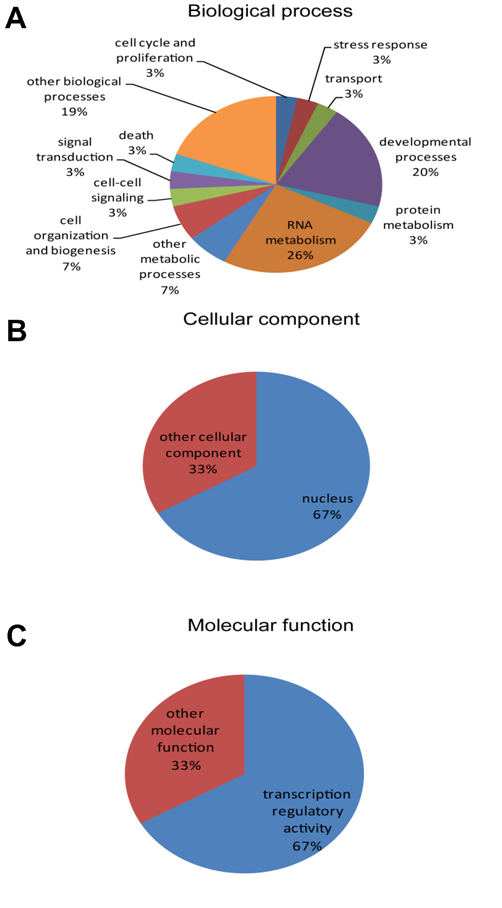
**

**Figure S2. Participation of the Two Key DRGs for MDD (SRK and JUN) in the MAPK Signaling Pathway**

Full schematic of the MAPK signaling pathway showing SRF and JUN (c-JUN) (highlighted in red).

**
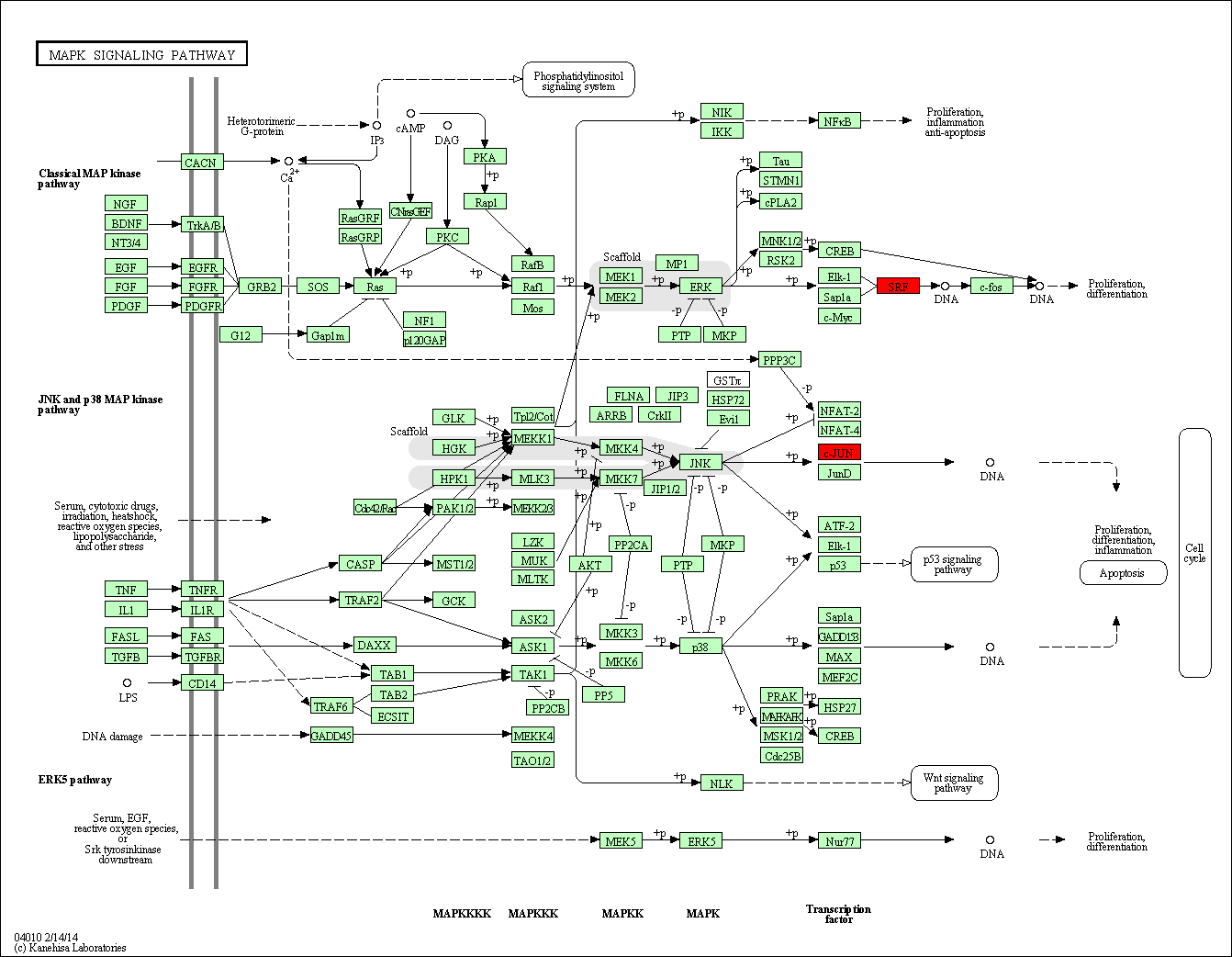
**
